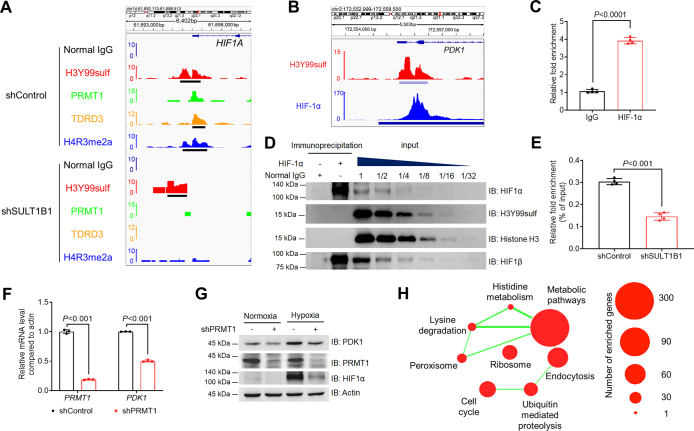# Histone H3Y99sulf regulates hepatocellular carcinoma responding to hypoxia

**DOI:** 10.1016/j.jbc.2024.105721

**Published:** 2024-02-02

**Authors:** Sibi Yin, Weixing Yu, Runxin Zhou, Xiao Zeng, Li Jiang, Yu Wang, Dingyuan Guo, Fuqiang Tong, Leya He, Jun Zhao, Yugang Wang

**Affiliations:** 1 Department of Biochemistry and Molecular Biology, School of Basic Medicine, Tongji Medical College, Huazhong University of Science and Technology, Wuhan, Hubei, China; 2 Department of Biochemistry and Molecular Biology, College of Basic Medicine, Jining Medical University, Jining, China; 3 Department of Neurosurgery, Renmin Hospital of Wuhan University, Wuhan, Hubei, China; 4 Department of Neurology, The Affiliated Nanhua Hospital, University of South China, Hengyang, China; 5 Department of Gastrointestinal Surgery, Tongji Hospital, Tongji Medical College, Huazhong University of Science and Technology, Wuhan, Hubei, China; 6 Department of Anatomy, School of Basic Medicine, Tongji Medical College, Huazhong University of Science and Technology, Wuhan, Hubei, China; 7 Cell Architecture Research Center, Huazhong University of Science and Technology, Wuhan, Hubei, China

**Keywords:** histone sulfation, regulation of gene transcription, hypoxia, glycolysis, PDK1

## Abstract

Histone H3 tyrosine-99 sulfation (H3Y99sulf) is a recently identified histone mark that can cross-talk with H4R3me2a to regulate gene transcription, but its role in cancer biology is less studied. Here, we report that H3Y99sulf is a cancer-associated histone mark that can mediate hepatocellular carcinoma (HCC) cells responding to hypoxia. Hypoxia-stimulated SNAIL pathway elevates the expression of *PAPSS2*, which serves as a source of adenosine 3′-phosphate 5′-phos-phosulfate for histone sulfation and results in upregulation of H3Y99sulf. The transcription factor TDRD3 is the downstream effector of H3Y99sulf-H4R3me2a axis in HCC. It reads and co-localizes with the H3Y99sulf-H4R3me2a dual mark in the promoter regions of *HIF1A* and *PDK1* to regulate gene transcription. Depletion of SULT1B1 can effectively reduce the occurrence of H3Y99sulf-H4R3me2a-TDRD3 axis in gene promoter regions and lead to downregulation of targeted gene transcription. Hypoxia-inducible factor 1-alpha and PDK1 are master regulators for hypoxic responses and cancer metabolism. Disruption of the H3Y99sulf-H4R3me2a-TDRD3 axis can inhibit the expression and functions of hypoxia-inducible factor 1-alpha and PDK1, resulting in suppressed proliferation, tumor growth, and survival of HCC cells suffering hypoxia stress. The present study extends the regulatory and functional mechanisms of H3Y99sulf and improves our understanding of its role in cancer biology.

Tyrosine sulfation is a recently identified type of histone modification that occurs at histone H3 tyrosine-99 residue (H3Y99sulf) in mammalian cells (1). The cytosolic sulfotransferase (SULT) superfamily member SULT1B1 is the installer of H3Y99sulf. It utilizes adenosine 3′-phosphate 5′-phos-phosulfate (PAPS) as the modification moiety donor and catalyzes H3Y99sulf on nascent histone H3 in cytosol. The H3Y99sulf-modified histone H3 then interacts with histone chaperons to be transported into the nucleus and deposited in transcription active regions in chromatin. The chromatin-H3Y99sulf mark can be exposed on the surface of subnucleosomal structures to recruit the arginine methyltransferase PRMT1, which deposits H4R3me2a to regulate gene transcription. While H3Y99sulf has been identified and studied in liver cancer cell lines, the role of H3Y99sulf in cancer biology of hepatocellular carcinoma (HCC) is unknown.

In the present study, we demonstrated that H3Y99sulf is a cancer-associated histone mark in HCC. Hypoxia signaling activates SNAIL pathway that upregulates the transcription of *PAPSS2*, which contributes PAPS for SULT1B1-mediated H3Y99 sulfation. Because hypoxia signaling occurs frequently in HCC, H3Y99sulf is significantly upregulated in tumor tissues. Besides, the H3Y99sulf-PRMT1-H4R3me2a axis is extended by the H4R3me2a-recruited TDRD3-TOP3B complex. It reads the H3Y99sulf-H4R3me2a dual mark in gene promoter regions, resolves transcription-associated R-loops, and facilitates transcription of downstream genes that regulate cancer metabolism and cellular responses to hypoxia, promoting the growth of HCC.

## Results

### H3Y99sulf is regulated by the SNAIL pathway in HCC

**Figure 1:**
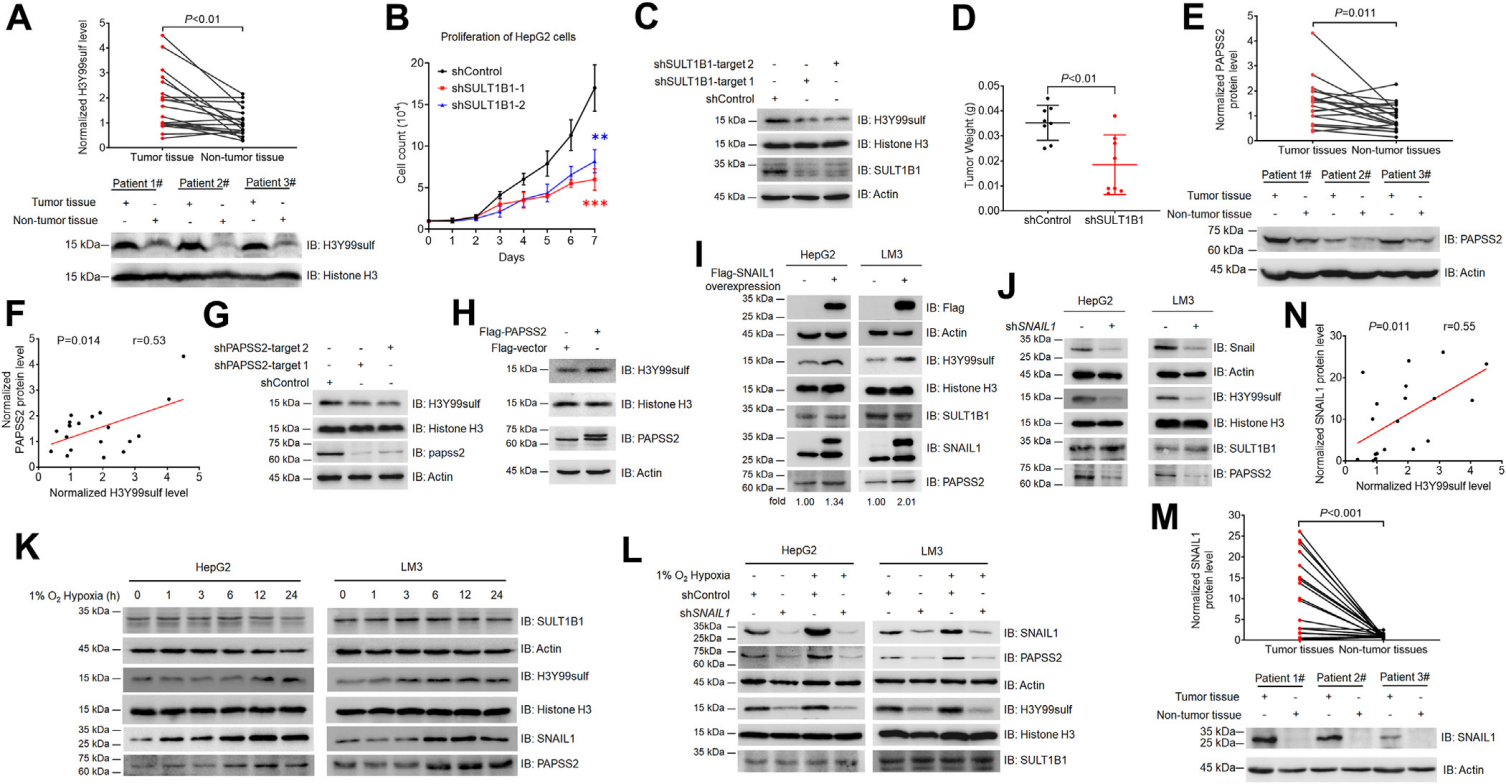
**H3Y99sulf is regulated by SNAIL pathway in HCC.***A*, the level of H3Y99sulf in clinical samples from HCC patients. The studied tumor tissues were collected from HCC patients. Normalized H3Y99sulf level in each tested sample was calculated as described in the Experimental procedures section. The relative levels of H3Y99sulf in the tumor and adjacent non-tumor tissues from the same patient were linked with *black lines* (n = 20). The representative images of immunoblotting assays are shown below. Two-sided *t*-tests were conducted to calculate the *p* value. *B* and *C*, SULT1B1 depletion reduced HCC cells proliferation. The cells were collected and counted daily for 7 days after the seeding of 10^4^ HepG2 cells (*B*). The data are presented as the means ± SD from four independent experiments (n = 4). Two-sided *t*-tests were conducted to calculate the *p* value, ∗∗*p* < 0.01, ∗∗∗*p* < 0.001. The effect of SULT1B1 depletion on H3Y99sulf level was examined by immunoblotting analyses with the indicated antibodies (*C*). Representative images of triplicate experiments are shown in the right-side panel. *D*, SULT1B1 depletion reduced xenograft tumor growth. HepG2 cells expressing shRNA against non-target and SULT1B1 were subcutaneously injected into athymic nude mice. Tumor weights were calculated. Data represent the means ± SD from nine independent experiments (n = 9). Two-sided *t*-tests were conducted to calculate the *p* value, ∗∗*p* < 0.01. *E*, PAPSS2 protein level in clinical samples from HCC patients. The studied tumor tissues were collected from HCC patients. Normalized PAPSS2 level in each tested sample was calculated as described in the Experimental procedures section. The relative levels of PAPSS2 in the tumor and adjacent non-tumor tissues from the same patient were linked with *black lines* (n = 20). The representative images of immunoblotting assays are shown below. Two-sided *t*-tests were conducted to calculate the *p* value. *F*, the correlation between PAPSS2 protein and H3Y99sulf levels in tumor samples. Normalized levels of PAPSS2 protein and H3Y99sulf were used in the correlation analysis. Two-sided *t*-tests were conducted to calculate the *p* value. Samples from 20 patients were studied (n = 20). *G* and *H*, the regulation of H3Y99sulf by PAPSS2 protein level in HCC cells. The H3Y99sulf level in cells with PAPSS2 knockdown (*G*) and ectopic overexpression (*H*) were tested by immunoblotting assays with the indicated antibodies. Representative images of triplicate experiments are shown. *I* and *J*, SNAIL1 protein and H3Y99sulf levels in HCC cells. The H3Y99sulf in HCC cells with SNAIL1 overexpression (*I*) and depletion (*J*) were tested by immunoblotting assays with the indicated antibodies. Representative images of triplicate experiments are shown. *K*, the effect of hypoxia on H3Y99sulf in HCC cells. The effects of hypoxia on H3Y99sulf levels in HCC cells were analyzed by immunoblotting assays with the indicated antibodies. Data represent three independent experiments. *L*, SNAIL1 mediates the response of H3Y99sulf to hypoxia. The effects of hypoxia on H3Y99sulf levels in HCC cells expressing shRNA against non-target and SNAIL1 were analyzed by immunoblotting assays with the indicated antibodies. Data represent three independent experiments. *M*, SNAIL1 protein levels in clinical samples from HCC patients. The studied tumor tissues were collected from HCC patients. Normalized SNAIL1 level in each tested sample was calculated as described in the Method section. The relative levels of SNAIL1 in the tumor and adjacent non-tumor tissues from the same patient were linked with *black lines* (n = 20). The representative images of immunoblotting assays are shown below. Two-sided *t*-tests were conducted to calculate the *p* value. *N*, the correlation between SNAIL1 protein and H3Y99sulf levels in tumor samples. Normalized levels of SNAIL1 protein and H3Y99sulf were used in the correlation analysis. Two-sided *t*-tests were conducted to calculate the *p* value. Samples from 20 patients were studied (n = 20).

**Supporting Figure S1:**
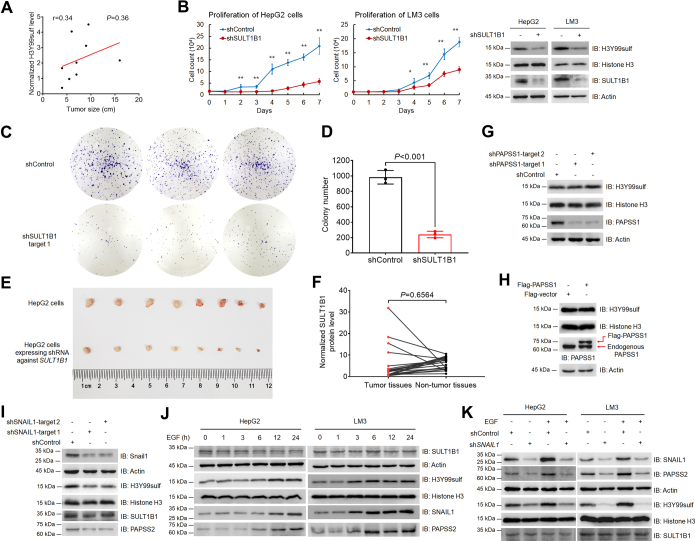


To understand whether H3Y99sulf correlates with HCC, we obtained tumor and tumor-adjacent tissues from HCC patients and analyzed the level of H3Y99sulf in human samples. The H3Y99sulf level in tumor tissue was significantly higher than that in normal liver tissue adjacent to a tumor in the same patient (Fig. 1*A*). The H3Y99sulf levels were proportionally correlated with the tumor sizes in poorly differentiated neoplasms (Fig. S1*A*), suggesting that H3Y99sulf associates with HCC development in patients. Depletion of SULT1B1, the installer of H3Y99sulf (1), reduced the level of H3Y99sulf and proliferation of HCC cell lines (Figs. 1, *B* and *C* and S1, *B*–*D*). The growth of a xenograft tumor generated by transplanting HCC cells in athymic nude mice was also suppressed by SULT1B1 loss (Figs. 1*D* and S1*E*). These results collectively suggest that H3Y99sulf might be a cancer-associated histone mark playing instrumental roles in HCC.

To understand the mechanism regulating H3Y99sulf in HCC tissues, we analyzed the protein level of SULT1B1 in HCC patients. No significant difference in SULT1B1 protein levels was found between the tumor tissues and their corresponding adjacent tissues in the same patients (Fig. S1*F*). PAPSS2 is the major enzyme in human liver producing PAPS for SULT1B1-mediated H3Y99 sulfation (2). We therefore examined and found that PAPSS2 protein level is upregulated in tumor tissues (Fig. 1*E*). And the H3Y99sulf levels were proportionally correlated with PAPSS2 protein levels in tumor tissues (Fig. 1*F*). Depletion of PAPSS2 effectively reduced H3Y99sulf in HCC cells (Fig. 1*G*). Ectopic overexpression of PAPSS2 upregulated H3Y99sulf in HCC cells (Fig. 1*H*). PAPSS1 is another human PAPS synthase isoform that majorly localized in nucleus (3, 4). Both depletion and overexpression of PAPSS1 did not change H3Y99sulf level in HCC cells (Fig. S1, *G* and *H*). These results illustrate that the increased H3Y99sulf level in tumor tissues might be caused by the upregulated PAPSS2 protein.

SNAIL1 is a transcriptional factor that can regulate the expression of *PAPSS2* (5). Overexpression of *SNAIL1* upregulated the levels of PAPSS2 protein and H3Y99sulf in HCC cells (Fig. 1*I*). Knockdown of *SNAIL1* in HCC cells decreased the levels of PAPSS2 protein and H3Y99sulf (Fig. 1*J* and S1*I*). Hypoxia and epidermal growth factor treatments can activate SNAIL1 pathway (6, 7). Both hypoxia and epidermal growth factor treatment upregulated the levels of SNAIL1 protein, PAPSS2 protein, and H3Y99sulf in HCC cells (Figs. 1*K* and S1*J*). The stimuli-increased H3Y99sulf were blocked by SNAIL1 depletion (Figs. 1*L* and S1*K*). In human HCC samples, the SNAIL1 protein levels in tumor tissues were significantly higher than those in their adjacent non-tumor tissues (Fig. 1*M*). Of noted, the H3Y99sulf levels in tumor tissues were proportionally correlated with SNAIL1 protein levels (Fig. 1*N*). Therefore, we conclude that the SNAIL1-PAPSS2 axis is the upstream mechanism regulating H3Y99sulf in HCC cells and tumor tissues.

### TDRD3 is a downstream effector of H3Y99sulf-H4R3me2a in HCC cells

**Figure 2:**
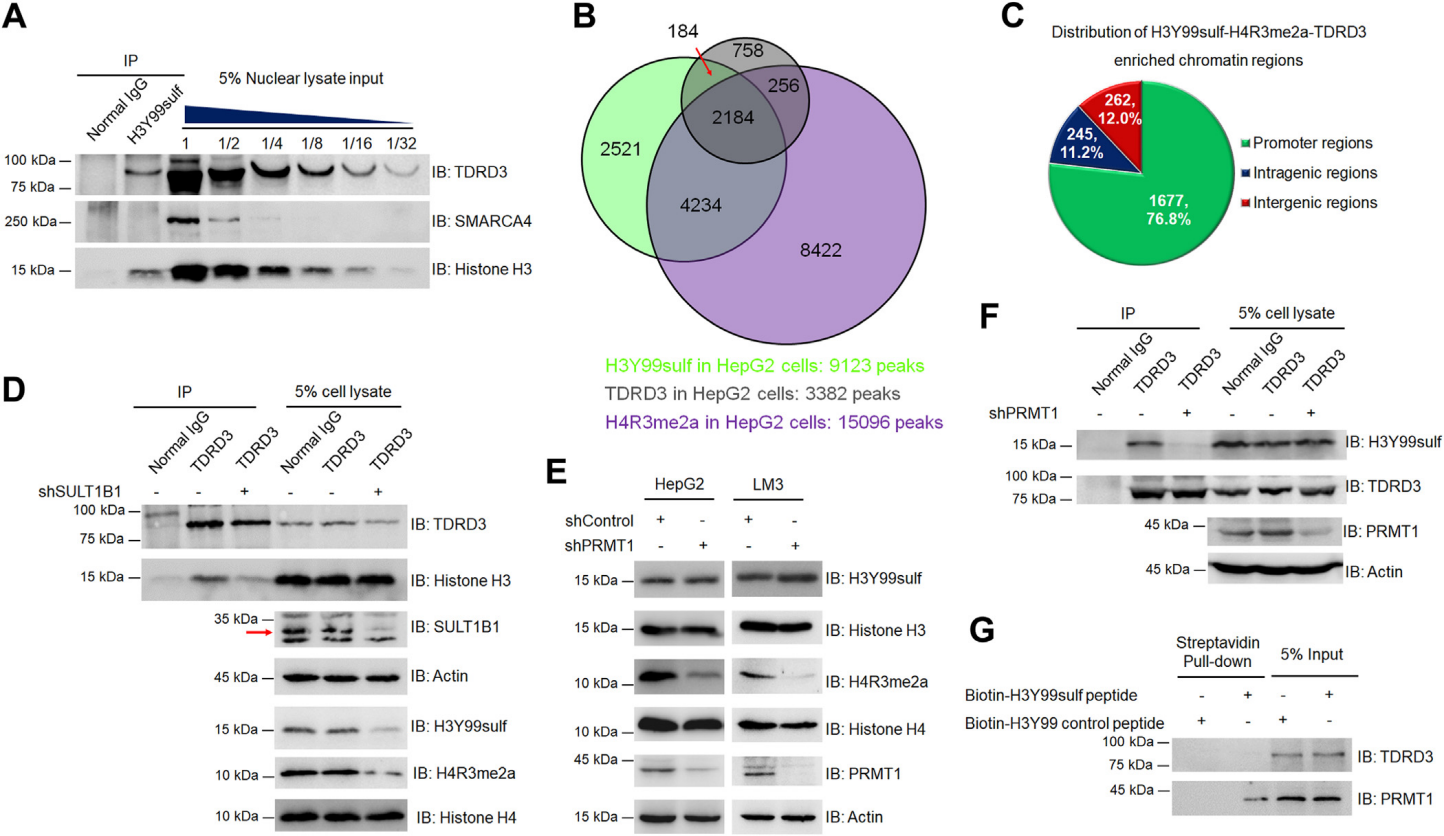
**TDRD3 is the downstream effector of H3Y99sulf-H4R3me2a.***A*, TDRD3 associates with H3Y99sulf-nucleosome. H3Y99sulf-nucleosome was immunoprecipitated from the formalin-fixed HepG2 cells using an anti-H3Y99sulf antibody. The immunoprecipitated proteins were re-crosslinked and analyzed by immunoblotting assays with the indicated antibodies. Representative images of triplicate experiments are shown. *B*, colocalization of TDRD3, H3Y99sulf and H4R3me2a in chromatin. Venn diagram showing the overlap of H3Y99sulf-enriched (*green*), H4R3me2a-enriched (*purple*), and TDRD3-binding chromatin regions (*grey*) in HepG2 cells (*p* < 0.01). *C*, genomic distribution of chromatin regions enriched by H3Y99sulf-H4R3me2a-TDRD3 axis in HepG2 cells. (fold enrichment ≥ 5, *p* < 0.001). *D*, SULT1B1 depletion reduced chromatin-binding of TDRD3. Endogenous TDRD3 was immunoprecipitated from HepG2 cells using an anti-TDRD3 antibody. The immunoprecipitated proteins were analyzed by immunoblotting assays with the indicated antibodies. Representative images of triplicate experiments are shown. *E*, the effect of PRMT1 depletion on the H3Y99sulf level in HCC cells. Immunoblotting assays were performed with the indicated antibody. Representative images of triplicate experiments are shown. *F*, the effect of PRMT1 depletion on the chromatin-binding of TDRD3. TDRD3 was immunoprecipitated from HepG2 cells expressing shRNA against non-target and PRMT1 by using anti-TDRD3 antibody, respectively. Immunoblotting assays were performed with the indicated antibodies. Representative images of triplicate experiments are shown. *G*, the interaction between TDRD3 and H3Y99sulf peptide. Nuclear extracts from cells were incubated with synthesized biotin-labelled peptides. Immunoblotting assays were performed with the indicated antibodies following the biotin-streptavidin pull-down. Representative images of triplicate experiments are shown.

H3Y99sulf co-localizes with H4R3me2a in gene promoter regions to regulate gene transcription (1). TDRD3 (Tudor domain containing 3) and SMARCA4 (a central component of the SWItch/Sucrose non-fermentable chromatin-remodeling complex) are effector molecules reading H4R3me2a to facilitate gene transcription (8, 9). To understand whether these H4R3me2a readers could serve as downstream effectors of the H3Y99sulf-H4R3me2a axis in HCC, we first performed immunoprecipitation assays and found the H3Y99sulf-enriched nucleosome associates with TDRD3 but not SMARCA4 (Fig. 2*A*), suggesting that H3Y99sulf might facilitate H4R3me2a selecting TDRD3 for downstream effects. We then performed cleavage under targets and tagmentation-sequencing (CUT&TAG-seq) analyses and identified 3382 TDRD3-binding chromatin regions (also named as peaks, fold enrichment (FE) ≥ 5, *p* ≤ 0.01), 70.0% of which (2368 peaks, FE ≥ 5, *p* ≤ 0.01) were also enriched by H3Y99sulf (Fig. 2*B*). We also noted that 92.2% of TDRD3-H3Y99sulf-enriched chromatin regions (2184 peaks, FE ≥ 5, *p* ≤ 0.01) were co-occupied by H4R3me2a (Fig. 2*B*). Most of the H3Y99sulf-H4R3me2a-TDRD3 chromatin regions (76.8%, 1677 out of 2184) were enriched in gene promoter regions (Fig. 2*C* and Table S1). Together with the transcriptional factor role of TDRD3, these findings illustrate the role of H3Y99sulf-H4R3me2a-TDRD3 axis in regulating gene transcription.

**Supporting Figure S2:**
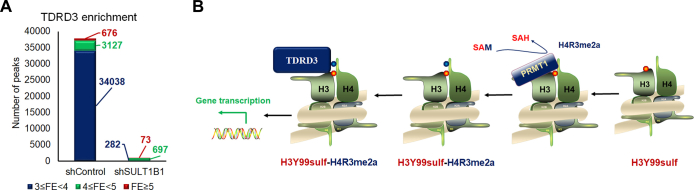


To validate the sequential mechanism of H3Y99sulf-H4R3me2a-TDRD3 axis, we disrupted H3Y99sulf by depleting SULT1B1 and found reduced histone binding of TDRD3 in HCC cells (Figs. 2*D* and S2*A*). Depletion of PRMT1 kept the H3Y99sulf level intact but decreased H4R3me2a and chromatin-binding of TDRD3 in HCC cells (Fig. 2, *E* and *F*). TDRD3 reads H4R3me2a but cannot directly interact with H3Y99sulf (Fig. 2*G*) (8). These findings illustrate that the PRMT1-catalyzed H4R3me2a is the upstream recruiting TDRD3 for chromatin-binding and the downstream that was directly regulated by H3Y99sulf (1). The H3Y99sulf-PRMT1-H4R3me2a-TDRD3 axis is sequentially established in gene promoter regions (Fig. S2*B*).

### H3Y99sulf-H4R3me2a-TDRD3 regulates *PDK1* in HCC cells

**Figure 3:**
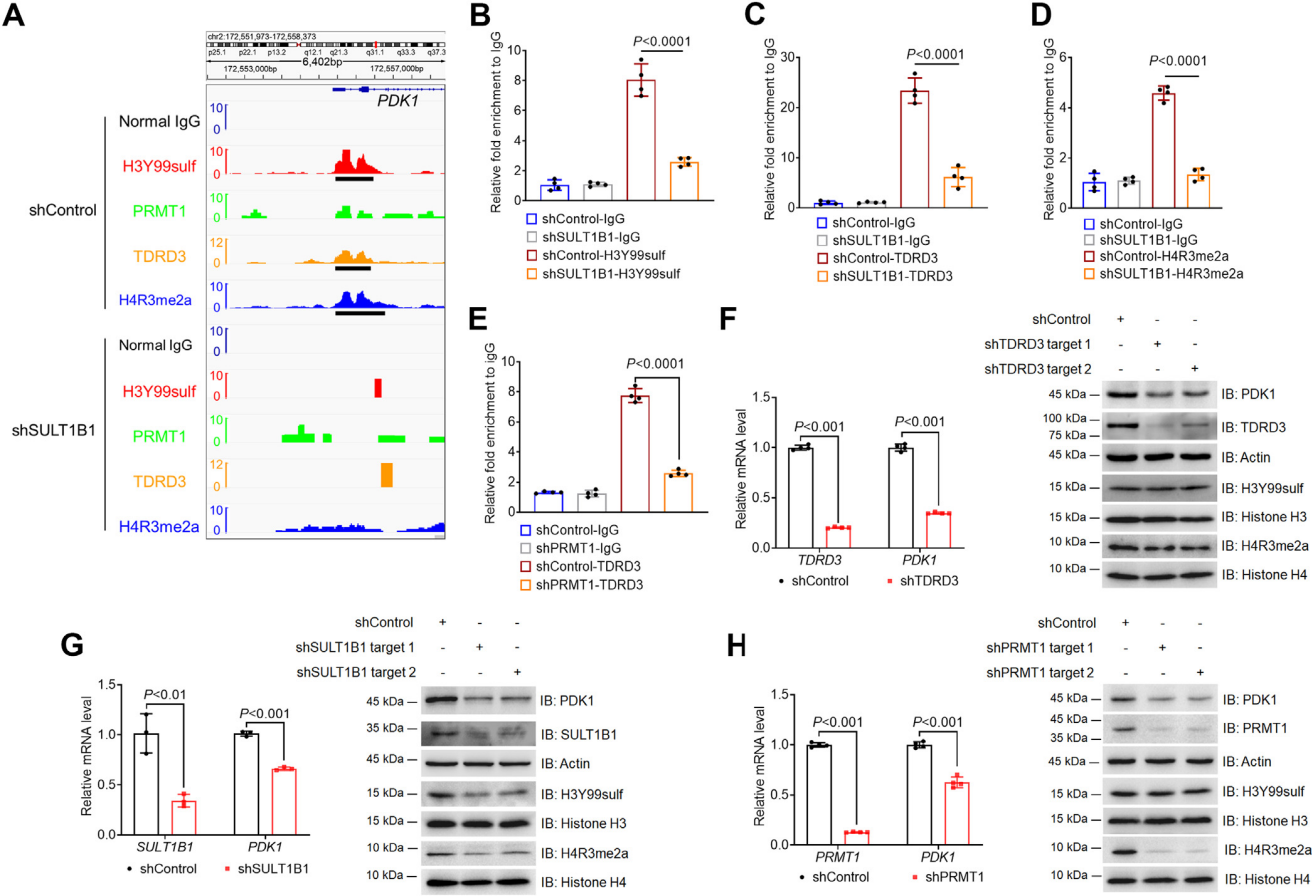
**H3Y99sulf-H4R3me2a-TDRD3 regulates PDK1 in HCC cells.***A*, colocalization of TDRD3, H3Y99sulf, PRMT1, and H4R3me2a in the promoter region of PDK1. In each row, the Y-axis represents fold enrichment level, and the *black bar* represents the peak detected by MACS2 (*p* < 0.01); one-sided *p* value was returned by MACS2 and calculated based on Poisson distribution. The enrichment of H3Y99sulf, PRMT1, and H4R3me2a at the promoter regions of PDK1 was obtained from our previously published work (1). *B*–*D*, enrichment of H3Y99sulf, TDRD3, and H4R3me2a in the promoter region of PDK1. Real-time PCR assays following cleavage under targets and tagmentation assays of H3Y99sulf (*B*), TDRD3 (*C*), and H4R3me2a (*D*) were performed. N = 4 biologically independent samples, two-sided *t*-tests were conducted to calculate the *p* value, and the data are presented as the means ± SD. *E*, the effect of PRMT1 depletion on the TDRD3-enrichment in the promoter region of PDK1. Real-time PCR assays following chromatin immunoprecipitation (ChIP) assays were performed. N = 4 biologically independent samples, two-sided *t*-tests were conducted to calculate the *p*-value, and the data are presented as the means ± SD. *F*–*H*, the effect of H3Y99sulf-H4R3me2a-TDRD3 on the expression of PDK1. Real-time PCR analysis of TDRD3 (*F*), SULT1B1 (*G*), and PRMT1 (*H*) were performed; n = 4 biologically independent samples, two-sided *t*-tests were conducted to calculate the *p* value, and the data are presented as means ± SD. Immunoblotting analysis was performed with indicated antibodies, and the results are shown on the right side of each part label. Representative images of triplicate experiments are shown.

**Supporting Figure S3:**
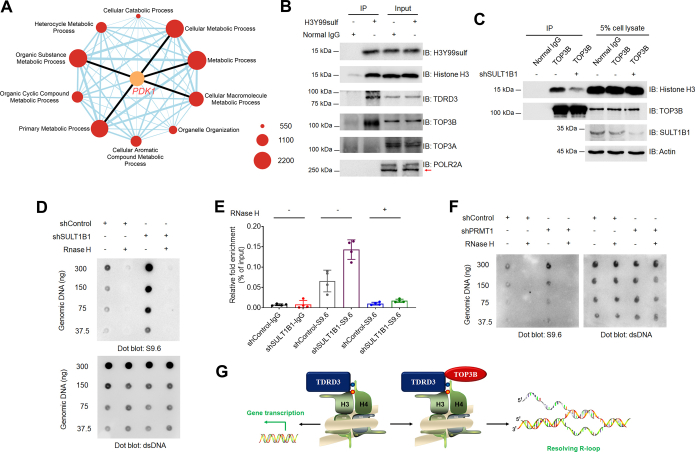


By performing gene ontology analysis, we found most of genes, whose promoter regions are occupied by the H3Y99sulf-H4R3me2a-TDRD3 axis, are enriched in metabolism-related processes (Fig. S3*A*). For instance, *PDK1* (encoding pyruvate dehydrogenase kinase 1) can be regulated by the H3Y99sulf-H4R3me2a axis (1). Its function is involved in multiple biological processes (Fig. S3*A*). Visualization of the CUT&TAG-seq results revealed colocalization of H3Y99sulf, PRMT1, H4R3me2a, and TDRD3 in its promoter region (Fig. 3*A*). Depletion of SULT1B1 turned off the signal and reduced H3Y99sulf, H4R3me2a, PRMT1, and TDRD3 in the promoter region of *PDK1* (Fig. 3, *A*–*D*). Depletion of PRMT1 stopped the signal flow from H3Y99sulf to H4R3me2a (1), so that the binding of TDRD3 to the promoter region of *PDK1* was reduced (Fig. 3*E*). TDRD3 depletion had no effect on the upstream H3Y99sulf and H4R3me2a (Fig. 3*F*), but it phenocopied the suppression of *PDK1* expression caused by SULT1B1 depletion and PRMT1 depletion (Fig. 3, *F*–*H*). These results demonstrate that the H3Y99sulf-H4R3me2a-TDRD3 axis localizes in the promoter region of *PDK1* to regulate its transcription in HCC cells.

In addition to regulating gene transcription, TDRD3 can recruit DNA topoisomerase 3β (TOP3B) to form the TDRD3-TOP3B complex (10), resolving transcription-associated R-loops to facilitate gene transcription. H3Y99sulf-immunoprecipitation assays identified that TDRD3-TOP3B complex can be enriched in H3Y99sulf-associated chromatin regions (Fig. S3*B*). Because H3Y99sulf-regulated H4R3me2a can recruit TDRD3 to chromatin (8), the binding of TOP3B to chromatin is mediated by TDRD3 (10). The interaction between TOP3B and chromatin was thereby suppressed by SULT1B1 depletion (Fig. S3*C*). TDRD3-TOP3B complex is an important mechanism for prevention of R-loop accumulation (10). Therefore, R-loop accumulation was observed in HCC cells with SULT1B1 depletion (Fig. S3*D*), including the intragenic regions of *PDK1* (Fig. S3*E*). Consistently, interrupting the H3Y99sulf-H4R3me2a axis by PRMT1 depletion phenocopied the R-loop accumulation that was observed in HCC cells with SULT1B1 depletion (Fig. S3*F*). Given the detrimental role of R-loop accumulation in gene transcription (10), we conclude that, in addition to directly regulating gene transcription, recruiting TOP3B to prevent R-loop accumulation is a supplementary mechanism for H3Y99sulf-H4R3me2a-TDRD3 axis facilitating gene transcription (Fig. S3*G*).

### H3Y99sulf mediates HCC cells responding to hypoxia

**Figure 4:**
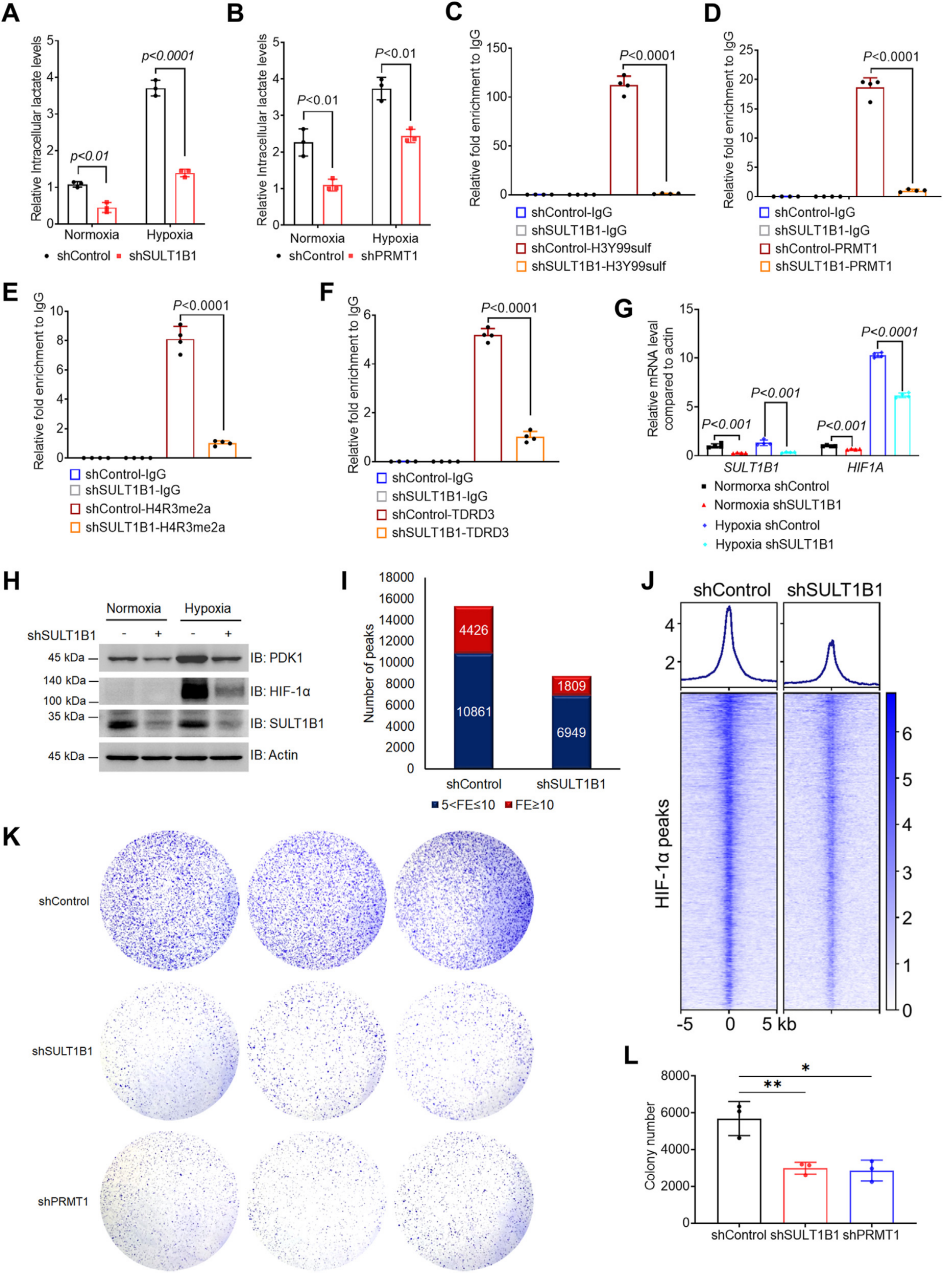
**H3Y99sulf mediates HCC cells responding to hypoxia.***A* and *B*, intracellular lactate levels in HCC cells. The intracellular lactate levels in HCC cells with SULT1B1 depletion (*A*) and PRMT1 (*B*) depletion were analyzed by using lactate quantification kit. n = 3 biologically independent samples, two-sided *t*-tests were conducted to calculate the *p*-value, and the data are presented as means ± SD. *C*–*F*, enrichment of H3Y99sulf, PRMT1, H4R3me2a, and TDRD3 in the promoter region of HIF1A. Real-time PCR assays following cleavage under targets and tagmentation assays of H3Y99sulf (*C*), PRMT1 (*D*), H4R3me2a (*E*), and TDRD3 (*F*) were performed. N = 4 biologically independent samples, two-sided *t*-tests were conducted to calculate the *p* value, the data are presented as the means ± SD. *G* and *H*, the expression of HIF1A in HCC cells. The mRNA and protein levels of HIF1A were analyzed by real-time PCR assays (*G*) and immunoblotting assays (*H*), respectively. The result of real-time PCR assays is presented as means ± SD. N = 4 biologically independent samples, and two-sided *t*-tests were conducted to calculate the *p* value. Representative images of triplicate immunoblotting assays are shown. *I* and *J*, the chromatin-occupancy of HIF-1α in HCC cells. The effect of SULT1B1 depletion on the occupancy of HIF-1α on chromatin (*p* < 0.01) (*I*) and gene promoter regions (*J*) are shown. *K* and *L*, the proliferation of HCC cells under hypoxia condition. The colony of HCC cells was visualized by crystal violet staining (*K*) and quantitatively analyzed 7 days after cells growing under hypoxia condition (*L*). The data are presented as the means ± SD from three independent experiments (n = 3). Two-sided *t*-tests were conducted to calculate the *p* value. HIF-1α, hypoxia-inducible factor 1-alpha.

PDK1 is a master regulator in glycolysis that functions as a central pathway in cancer metabolism (11, 12). Because the H3Y99sulf-H4R3me2a-TDRD3 axis controls *PDK1* expression (Fig. 3, *A*–*D*), SULT1B1 depletion turned off the H3Y99sulf-H4R3me2a-TDRD3 axis and reduced *PDK1* expression (Fig. 3*G*). Consequently, lactate production was reduced in HCC cells with SULT1B1 depletion (Fig. 4*A*), indicating suppressed glycolysis. Blocking the H3Y99sulf-H4R3me2a axis by PRMT1 depletion also decreased lactate production in HCC cells (Fig. 4*B*). These findings illustrate that H3Y99sulf-regulated *PDK1* expression controls glycolysis.

Hypoxia frequently occurs in most solid tumors and induces hypoxia-inducible factor 1-alpha (HIF-1α), which transactivates metabolic genes that regulate glycolysis (13, 14). By performing CUT&TAG-sequencing assays, we identified colocalization of H3Y99sulf, H4R3me2a, PRMT1, and TDRD3 in the promoter region of *HIF1A* (encoding HIF-1α) in HCC cells (Fig. S4*A*). SULT1B1 depletion reduced the occurrence of the H3Y99sulf-H4R3me2a-TDRD3 axis in the promoter region of *HIF1A* (Figs. 4, *C*–*F* and S4*A*) and the expression of *HIF1A* in HCC cells, regardless in normoxia and hypoxia environments (Fig. 4, *G* and *H*). As a consequence, the chromatin occupancy of HIF-1α in cells suffering hypoxia stress was decreased by SULT1B1 depletion (Fig. 4, *I* and *J*).

The transcription of *PDK1* can be regulated by HIF-1α (13). By performing chromatin immunoprecipitation assays, we unexpectedly found that HIF-1α and H3Y99sulf occupy the same promoter region of *PDK1* (Fig. S4, *B* and *C*). However, histone is dispensable for HIF-1α binding to chromatin (15). No interaction was found between the H3Y99sulf-modified histone H3 and HIF-1α (Fig. S4*D*), suggesting no direct cross-talk between H3Y99sulf and HIF-1α, despite their co-localization in the promoter region of *PDK1*. The binding of HIF-1α to the promoter region of *PDK1* was reduced in the SULT1B1-depleted cells (Fig. S4*E*). Together with the regulatory role of H3Y99sulf-H4R3me2a in the transcription of *PDK1*, we conclude that the suppressed transcription of *PDK1* in the H3Y99sulf-H4R3me2a axis disrupted HCC cells, which was synergistically caused by the reduced HIF-1α binding and H3Y99sulf-H4R3me2a-TDRD3 axis in its promoter region (Figs. 4, *G* and *H* and S4, *A*, *F*, and *G*). As a consequence, lactate production was also synergistically inhibited in HCC cells with a disrupted H3Y99sulf-H4R3me2a axis (Fig. 4, *A* and *B*).

Enhanced glycolysis is important for cancer cells surviving and proliferating in hypoxic stress (16). Disrupting H3Y99sulf by SULT1B1 depletion or cutting off the H3Y99sulf-H4R3me2a axis by PRMT1 depletion reduced survival of HCC cells in hypoxia (Fig. 4, *K* and *L*). In addition to *PDK1*, HIF-1α can regulate a broad transcriptome in cells suffering from hypoxia (17). Disruption of H3Y99sulf-H4R3me2a reduced the occupancy of HIF-1α in a number of gene promoter regions that participate in important biological processes, such as metabolism, lysine degradation, cell cycle, and endocytosis (Fig. S4*H* and Table S2). Therefore, disruption of SULT1B1-mediated H3Y99sulf might severely influence the homeostasis of HCC cells and result in poor survival of HCC cells in an hypoxia environment. These findings demonstrate that the H3Y99sulf-H4R3me2a-TDRD3 axis can regulate the transcription of central genes, including *PDK1* and *HIF1A*, and harness the response of HCC cells to hypoxia environment, supporting the survival and growth of HCC cells.

## Conclusion and discussion

Histone H3Y99 sulfation is a recently identified histone mark that is modified in cytosol and functions in nucleus (1). The present study demonstrates that H3Y99sulf is a cancer-associated histone mark that mediates HCC cells responding to hypoxia. SNAIL1-induced PAPSS2 is the upstream of SULT1B1-mediated H3Y99sulf. It can be activated to upregulate H3Y99sulf in HCC cells in response to hypoxia. TDRD3 is the downstream effector of the H3Y99sulf-H4R3me2a axis, regulating gene transcription *via* three layers of mechanism. First, because TDRD3 is a transcription factor, the recruitment of TDRD3 by H3Y99sulf-H4R3me2a dual mark can directly activate gene transcription. Second, the H3Y99sulf-H4R3me2a-TDRD3-TOP3B axis can prevent transcription-associated R-loop accumulation to secure highly-efficient transcription. Third, besides directly activating gene transcription, the H3Y99sulf-H4R3me2a-TDRD3 axis can regulate the expression of transcription factors, such as HIF-1α, which can also harness gene transcription.

The present study refines our understandings about histone sulfation in mammals. It also extends the spectrum of cancer-associated histone marks and the repertoire of mechanisms underlying cancer biology. However, histone modifications are linked to multiple biological processes in cancer and other diseases (18, 19). The regulatory role of H3Y99sulf-H4R3me2a-TDRD3 in the transcription of *PDK1* and *HIF1A*, as well as the function in HCC, are illustrative instances of this newly identified histone mark. More mechanistic and functional studies of H3Y99sulf in different physiologies and pathophysiologies are expected.

## Experimental procedures

The human sample studies were conducted in accordance with the Declaration of Helsinki. Detailed information about experimental procedures is included in Supporting Information file.

## Data availability

The data that support the findings of this study are included in the main article and Supplementary Information files. The CUT&Tag-seq and chromatin immunoprecipitation-seq data have been deposited in the Genome Sequence Archive for Human with the accession numbers subHRA007656 and HRA003214.

## Supporting information

This article contains supporting information (20, 21, 22, 23, 24, 25, 26, 27).

## Conflict of interest

The authors declare that they have no conflicts of interest with the contents of this article.

## Figures and Tables

**Supporting Figure S4:**