# Supplementary material for: Histone H3Y99sulf regulates hepatocellular carcinoma responding to hypoxia
Source: J Biol Chem. 2024 Feb 2;300(3):105721. doi: 10.1016/j.jbc.2024.105721 (PMC10910123; doi:10.1016/j.jbc.2024.105721)
Supplement: Supporting figure and table legends [file mmc6.docx]

**Supporting Information figure legends**

**Fig S1. H3Y99sulf is regulated by Snail pathway in HCC**

(**A**), The correlation between H3Y99sulf level and tumor size in human samples. The studied tumor tissues were collected from HCC patients. Normalized levels of H3Y99sulf and tumor size in each tested sample were used in the correlation analysis. Two-sided *t*-tests were conducted to calculate the *P*-value. Samples from 9 low-differentiated HCC patients were studied (n=9).

(**B**) SULT1B1 depletion reduced proliferation of HCC cells. A total of 10^4^ HepG2 cells and LM3 cells expressing shRNA against non-target and SULT1B1 were plated. The cells were collected and counted daily for 7 days. The data are presented as the means±s.d. from four independent experiments (n=4). The effect of shRNA targets against SULT1B1 on H3Y99sulf level was examined by immunoblotting analyses with the indicated antibodies. Representative images of triplicate experiments are shown in the right-side panel. Two-sided *t*-tests were conducted to calculate the *P*-value, ***P*<0.01, ****P*<0.001.

(**C**) and (**D**), The effect of SULT1B1 depletion on HCC cells proliferation. A total of 10^3^ HepG2 cells with or without SULT1B1 depletion were plated. The colonies were visualized crystal violet staining (**C**) and counted quantitatively analyzed by ImageJ software 7 days after cell-seeding (**D**). The data are presented as the means±s.d. from three independent experiments (n=3). Two-sided *t*-tests were conducted to calculate the *P*-value.

(**E**), SULT1B1 depletion reduced xenograft tumor growth. HepG2 cells expressing shRNA against non-target and SULT1B1 were subcutaneously injected into athymic nude mice. Tumor collected and measured three weeks after transplantation.

(**F**), SULT1B1 protein levels in human samples. The studied tumor tissues were collected from HCC patients. Normalized SULT1B1 level in each tested sample was calculated as described in the method section. The relative levels of SULT1B1 in the tumor and adjacent non-tumor tissues from the same patient were linked with black lines (n=20). The representative images of immunoblotting assays are shown below. Two-sided *t*-tests were conducted to calculate the *P*-value.

(**G**), Effect of PAPSS1 depletion on H3Y99sulf in HCC cells. Immunoblotting assays were performed with the indicated antibodies. Data represent three independent experiments.

(**H**), Effect of PAPSS1 overexpression on H3Y99sulf in HCC cells. Immunoblotting assays were performed with the indicated antibodies. Data represent three independent experiments.

(**I**), Effects of SNAIL1 depletion. Immunoblotting assays were performed with the indicated antibodies. Data represent three independent experiments.

(**J**), The effect of EGF treatment on H3Y99sulf in HCC cells. The levels of H3Y99sulf in HCC cell lines with EGF treatment were analyzed by performing immunoblotting assays with the indicated antibodies. Data represent three independent experiments.

(**K**), The effect of SNAIL1 depletion in HCC cells treated by EGF. The H3Y99sulf level in HCC cell lines with EGF treatment were analyzed by performing immunoblotting assays with the indicated antibodies. Data represent three independent experiments.

**Fig S2. TDRD3 is the downstream effector of H3Y99sulf-H4R3me2a**

(**A**), The effect of SULT1B1 depletion on the distribution of TDRD3 on chromatin. The number of TDRD3-binding chromatin regions in HepG2 cells expressing shRNA against non-target and *SULT1B1* are shown, respectively. The TDRD3 peaks (*P*<0.01) were catalogized by the degree of fold enrichment (3≤FE<4, 4≤FE<5, FE≥5), one-sided p-value was returned by MACS2 and calculated based on Poisson Distribution.

(**B**), Scheme of TDRD3 reading H3Y99sulf-H4R3me2a dual mark.

**Fig S3. H3Y99sulf-H4R3me2a-TDRD3 regulates *PDK1* in HCC cells**

(**A**), Gene enrichment of H3Y99sulf-H4R3me2a-TDRD3 associated genes. Pathways that might be regulated by H3Y99sulf-H4R3me2a-TDRD3 enriched genes are shown in nodes (Fold enrichment >4, *P*<0.01; For pathway enrichment, *P*<0.01). The node size reflects the number of H3Y99sulf-H4R3me2a-TDRD3 enriched genes in the pathway. Edges represent the number of genes is shared between the pathways. The position of *PDK1* in the pathway network is shown.

(**B**), The bindings of TOP3B and TDRD3 to H3Y99sulf. H3Y99sulf-nucleosome was immunoprecipitated from the formalin-fixed HepG2 cells using an anti-H3Y99sulf antibody. The immunoprecipitated proteins were re-crosslinked and analyzed by immunoblotting assays with the indicated antibodies. Representative images of triplicate experiments are shown.

(**C**), The chromatin binding of TOP3B is reduced by SULT1B1 depletion. TOP3B was immunoprecipitated from the cultured HepG2 cells expressing shRNA against non-target and SULT1B1, respectively. Immunoblotting assays were performed with the indicated antibodies. Representative images of triplicate experiments are shown.

(**D**), R-loop accumulation tests. Genomic DNA was purified from the HepG2 cells expressing shRNA against non-target and SULT1B1, respectively. The R-loop was analyzed by using immunoblotting assays with the R-loop specific antibody S9.6. The genomic DNA in each sample was treated with RNaseH to serve as negative control. The same set of samples was incubated with an antibody against dsDNA to examine the sample loading amount. Representative images of triplicate experiments are shown.

(**E**), R-loop accumulation in the intragenic region of *PDK1*. The R-loop in the coding region of *PDK1* was assessed by performing qDRIP assays. The two-sided student *t*-tests were conducted to compute *P*-value. The data are presented as the means±s.d. from four independent experiments (n=4). ***P*<0.01.

(**F**), R-loop accumulation tests in PRMT1 depleted cells. Genomic DNA was purified from the HepG2 cells expressing shRNA against non-target and PRMT1, respectively. The R-loop accumulation was analyzed using immunoblotting assays with the R-loop specific antibody S9.6. The genomic DNA in each sample was treated with RNaseH to serve as negative control. The same set of samples was incubated with an antibody against dsDNA to examine the sample loading amount. Representative images of triplicate experiments are shown.

(**G**), Scheme of H3Y99sulf-H4R3me2a recruited TDRD3 form complex with TOP3B to prevent accumulation of R-loops.

**Fig S4. H3Y99sulf mediates HCC cells responding to hypoxia**

(**A**), Colocalization of H3Y99sulf, PRMT1, H4R3me2a and TDRD3 in the promoter region of *HIF1A*. In each row, the Y-axis represents fold enrichment level and the black bar represents the peak detected by MACS2 (*P*<0.01), one-sided *P*-value was returned by MACS2 and calculated based on Poisson distribution.

(**B**), Colocalization of H3Y99sulf and HIF-1α in the promoter region of *PDK1*. Combined view of CUT&TAG-seq and ChIP-seq analyses revealing H3Y99sulf and HIF-1α occupy the same region in *PDK1* promoter.

(**C**), Validation of the occupancy of HIF-1α in H3Y99sulf-enriched region in the promoter of *PDK1*. Real-time PCR assays following HIF-1α-ChIP experiment. The H3Y99sulf-enriched promoter region of *PDK1* was previously identified. The data are presented as the means±s.d. from four independent experiments (n=4). ***P*<0.01.

(**D**), Interaction between HIF-1α and histone H3 in HCC cells. HIF-1α was immunoprecipitated from the cultured HepG2 cells using an anti-HIF-1α antibody. Immunoblotting assays were performed with the indicated antibodies. Representative images of triplicate experiments are shown.

(**E**), Enrichment of HIF-1α in the promoter region of *PDK1*. Real-time PCR assays following ChIP assays with indicated antibodies were performed, n=4 biologically independent samples, two-sided *t*-tests were conducted to calculate the *P*-value, the data is presented as the means±s.d.

(**F**) and (**G)**, The expression of *HIF1A* in PRMT1 depleted cells. The mRNA and protein levels of *HIF1A* were analyzed by Real-time PCR assays (**F**) and immunoblotting assays with indicated antibodies (**G**), respectively. The result of Real-time PCR assays is presented as the means±s.d, n=4 biologically independent samples, two-sided *t*-tests were conducted to calculate the *P*-value. Representative images of triplicate immunoblotting assays are shown.

(**H**), HIF-1α-enriched pathways that were significantly suppressed by SULT1B1 depletion. The fold enrichment of HIF-1α below 5 (FE<5) was defined as loss of HIF-1α binding. Genes whose promoter regions lose HIF-1α binding were collected for KEGG analysis (Modified Fisher Exact P-value, *P*<0.01). The red node size reflects the number of genes whose promoter region lost HIF-1α binding in the pathway. Edges represent more than one gene are shared between the pathways.
