# Supplementary material for: Histone H3Y99sulf regulates hepatocellular carcinoma responding to hypoxia
Source: J Biol Chem. 2024 Feb 2;300(3):105721. doi: 10.1016/j.jbc.2024.105721 (PMC10910123; doi:10.1016/j.jbc.2024.105721)
Supplement: Supporting information [file mmc7.docx]

**Experimental procedures**

**Materials**

Detailed information about antibodies used in the experiments is listed in Table S3. Streptavidin-conjugated agarose beads and RNase H were purchased from Beyotime. Polyethylenimine (Linear) was purchased from Yeasen Biotechnology. Biotion-H3Y99(biotin-QSAAIGALQEASEAYLVGLFEDTNLCAIHAK) and sulfated biotin-H3Y99(Biotin-QSAAIGALQEASEAY[sulfation]LVGLFEDTNLCAIHAK) peptides were obtained from Thermo Fisher Scientific. Phosphatase inhibitor cocktail I, phosphatase inhibitor cocktail II, deacetylase inhibitor cocktail, and protease inhibitor cocktail were purchased from MedChemExpress.

**Cell culture**

HepG2, LM3,293T cells were obtained from ATCC. All cell lines were confirmed to be

mycoplasma-free. HepG2, LM3,293T cells were cultured in DMEM (SH30285.01, Hyclone) supplemented with 10% fetal bovine medium (164210-50, Pricella) and 1% penicillin–streptomycin at 37 °C in a humidified CO_2_ chamber.

**Hypoxia treatment**

For hypoxia treatment, cells were serum starved for 12 hours prior to hypoxia treatment for 24h in a hypoxic chamber. To avoid reoxygenation, RNA, protein, and metabolite samples were collected within the hypoxic workstation.

**DNA constructs**

All plasmids were constructed using standard molecular biology technology. PCR-amplified human PAPSS2, Snail, and TOP3B were cloned into pHAGE/puro (+)-Flag or pHAGE/neo(+)-Flag vector. The sequences of shRNAs interfering the mRNA level of SULT1B1, PRMT1, TDRD3, PAPSS1, PAPSS2, and SNAIL were listed in Table S4.

**Lentivirus production and infection**

A total of 2× 10^6^ HEK293T cells were transfected with the constructed plasmids above, packaging plasmid psPAX2 and the envelope plasmid pMD2.G with PEI (Polyethylenimine) transfection reagent (40815ES03, Yeasen Biotechnology) according to manufacturer’s instruction. The medium was changed at 12h post-transfected, then the supernatant containing lentiviruses was harvested at 36h post-transfected and 60h post-transfected. For lentivirus infection, 1ml of virus-containing medium was used for 6 × 10^4^cells in the presence of 10 ug/ml polybrene (C0351, Beyotime).

**Cell proliferation assay**

For cell proliferation assay, cells were seeded in appropriate culture dishes. The cell numbers of each group were counted every day for 7 d. For the cell colony formation assay, cells (1×10^4^) were seeded in six-well plates and cultured for 7 d. The colonies were determined by crystal violet staining.

**Intracellular lactate measurement**

Intracellular lactate was measured with a lactic acid assay kit (BC2230, Solarbio) according to the manufacturer’s instructions. Briefly, 2 x 10^6^ cells were collected and washed with cold PBS, and then lysed and extracted with the corresponding buffer in the kit. The absorbance of lactate was measured at 570nm and the content of lactate was calculated according to a standard curve. The values were normalized to the protein concentration.

**Western blot**

Extraction of proteins from cultured cells was performed with RIPA lysis buffer (50 mM Tris (pH 8.0), 150 mM NaCl, 1% Triton X-100, 1% sodium deoxycholate,0.1% SDS) supplemented with protease inhibitor cocktail (MedChemExpress) and phosphatase inhibitor cocktail (MedChemExpress). The protein concentrations were measured by Bradford Protein Assay Kit (P0006C, Beyotime). Equal amounts of protein were separated by SDS-PAGE and transferred to NC membranes.

**RNA extraction and real-time PCR**

Total RNA of cells was isolated with the RNAsimple Total RNA Kit (DP419, TIANGEN). 500 ng of total RNA was reverse-transcribed into cDNA with ABScript III RT Master Mix for qPCR with gDNA remover (RK20429, ABclonal technology). Quantitative real-time PCR was performed with 2 × Universal SYBR Green Fast qPCR Mix (RK21203, Abclonal Technology). The procedure parameters were set at 95°C for 3 min, then 95°C for 5 s, and 60°C for 32s (40 cycles, in total). ACTB was used as a control. The primers used for real-time PCR are listed in Table S5.

**Biotin pull-down assays**

Comparable amounts of biotin-H3Y99 control peptides and biotin-H3Y99sulf peptides were incubated with cell lysate overnight. Mixtures were incubated with streptavidin beads for another 4 hours and then washed 5 times, subjected to SDS-PAGE, and immunoblotted with the indicated antibodies.

**Immunoprecipitation and Immunoblotting assays**

Protein were extracted from cultured cells using the buffer that described previously^1^. Immunoprecipitation and immunoblotting analyses with the indicated antibodies were performed as described previously ^1^. The fix immunoprecipitation assays were performed with a modified protocol. Briefly, the steps of cross-links and immunoprecipitation for fix immunoprecipitation were performed using a Simple ChIP Enzymatic Chromatin IP Kit (Cat#9003, Cell Signaling Technology, MA, USA), and protease inhibitor cocktail instead of protease K were used in reversal cross-links. The results from immunoblotting assays were visualized and recorded using Tanon chemiluminescence imaging system (Tanon 5200, Shanghai, China).

**Chromatin Immunoprecipitation (ChIP) assay**

ChIP assays were performed with SimpleChIP Enzymatic Chromatin IP Kit (9003, Cell Signaling Technology) according to the manufacturer’s instructions. Briefly, 1.5 x 10^7^ cells were stimulated by hypoxia for 24h and then were cross-linked with 1% formaldehyde for 10 minutes. Then cells were washed and scraped into cold PBS. After centrifugation, cells were resuspended in lysis buffer to obtain nuclei. The released nuclei were lysed and sonicated to shear chromatin to 200–500 bp in nuclear lysis buffer. DNA was digested to length of approximately 150-900bp with Micrococcal Nuclease. After diluting with ChIP buffer to appropriate volume, the cross-linked chromatin was incubated with the indicated antibody overnight. Protein G magnetic beads were added and incubated for another 2h. After washing, elution, and reverse-cross-linking, the ChIP DNA was purified and sequenced on Illumina platforms with PE150 strategy in Novogene Bioinformatics Technology, according to effective library concentration and data amount.

**CUT & TAG assay**

CUT and Tag assays were performed with the Vazyme Hyperactive In-Situ ChIP Library Prep Kit (TD902, Vazyme) according to the manufacturer’s instructions. Briefly, 1×10^6^ cells were collected, washed, and incubated with ConA beads for 10 min at room temperature, then each group was incubated with the indicated antibody (1:50 dilution) at room temperature for 2 h. After binding to ConA beads, Mouse Anti-rabbit IgG secondary antibody was added and incubated at room temperature (1:100 dilution) for 1 h. Then the beads were washed and incubated with 0.04 μM pA-Tn5 Transposon. After recovering the fragmented DNA, TruePrep Index Kit V2 for Illumina (TD202 Vazyme) was used to construct PCR library (15 cycles), and VAHTS DNA clean beads (N411–01-AA, Vazyme) were used to purify DNA. The DNA products were performed sequencing analysis on the Illumina platform (NovaSeq 6000) with PE150 strategy in Novogene Bioinformatics Technology, according to effective library concentration and data amount required.

**Xenograft models**

All mouse experiments were proved by the Institutional Animal Care and Use Committee of Huazhong University of Science and Technology. The 4-week-old female athymic nude mice were purchased from Hubei Bainte Biological Technology Co., Ltd. Mice were allowed to acclimatize for one week in a pathogen-free environment. For xenograft models, 5×10^6^ HepG2 cells with or without SULT1BI knock-down were injected into the subcutaneous region of nude mice that were randomly grouped. After the cancer cells implantation, mice were monitored every day. Xenograft tumors were collected from sacrificed mice three weeks after HCC cells transplantation.

**Patient samples**

This study was approved by the Ethics Committee of Tongji Medical College. The study was conducted according to the principles of the Declaration of Helsinki. Twenty pairs of fresh HCC tissues and adjacent benign tissues were collected after surgical resection from Tongji Hospital of Tongji Medical College. All the patients involved in the donation provided written informed consents. The differentiation statuses were graded according to the method of Edmondson and Steine.

The protein levels and H3Y99sulf level in each clinical sample were normalized for quantitative analysis. The levels of H3Y99sulf and histone H3 in each sample were analyzed by using immunoblotting assays with the antibody against H3Y99sulf and histone H3, respectively. The signal intensity of each immunoblotting assay was quantified by ImageJ software. The ratio of H3Y99sulf intensity to histone H3 intensity was calculated as the relative intensity of H3Y99sulf in each tested sample. Each immunoblotting assay contains a standard sample. The normalized H3Y99sulf of each sample is the ratio of relative intensity of H3Y99sulf in each tested sample to the one in standard sample. The way of target protein normalization is similar. The relative intensity of target proteins in each tested sample was calculated as the ratio of target protein against Actin.

**R-loop detection**

Genomic DNA was extracted with a Tissue DNA kit (D3396, OMEGA) from 5x10^6^ HepG2 cells and normalized to 300ng/ul with nanodrop (Thermo Fisher Scientific), One microliter of genomic DNA of each group was spot on Hybond-N + membrane (YA1760, Solarbio), UV-crosslinked (120mJ/cm^2^) with Ultraviolet crosslinkers (CL-1000L, analytik-jena,), blocked with 5% BSA in TBST and incubated with S9.6 antibody overnight at 4°C. After washing with PBS-Tween and incubation with secondary antibody, the signal was detected.

**DRIP assays**

Briefly, nucleic acids were extracted from HepG2 cells using Tissue DNA Kit (D3396, OMEGA). The harvested nucleic acids were digested for 24 h at 37°C using a restriction enzyme cocktail (50 units/100 μg nucleic acids, each of XhoI, EcoRI, HindIII, and XbaI) in the New England Biolabs CutSmart buffer. Digested DNA was purified by Cycle Pure Kit (D6492, OMEGA) followed by treatment with or without RNase H (D7089, Beyotime) overnight at 37°C. RNA: DNA hybrids(4ug) were immunoprecipitated using 10 μg of S9.6 antibody and 30 μl of protein A/G beads at 4°C for 2 h in IP buffer. The beads were then washed four times with IP buffer for 10 min at 4°C, and the nucleic acids were eluted with elution buffer (50 mM Tris–HCl, pH8.0, 10 mM EDTA, 0.5% SDS, and 70 μg protease K) at 55°C for 1 h. Immunoprecipitated DNA was then cleaned up by Cycle Pure Kit (D6492, OMEGA). Using the website (http://rloop.bii.a-star.edu.sg/) to find an R-LOOP location and design primers around that location. The Quantitative PCR procedure was described above.

**Bioinformatic analysis**

Sequencing reads from the ChIP-seq and CUT&TAG-seq raw data were aligned to the reference genome using Bowtie2 2.2.5^2^. The resulted alignment files were sorted and indexed using SAMtools 1.6.0^3^. To reduce amplification bias, PCR duplicates were removed by using Picard 2.26.6 (<https://broadinstitute.github.io/picard/>). Peak calling was performed by using Macs2 2.2.7.1^4^ and statistically assessed by modified Fisher exact *P*-value.

Pathway enrichment was analyzed by utilizing the Gene Ontology Biological Process (GO BP) database on the platform of DAVID^5,6^_._ Fisher’s exact test was used to determine the *P*-values of the pathway enrichments. The results were ranked based on *P*-values. Top 10 pathways were selected for visualization by employing the Enrichment Map plugin within Cytoscape^7,8^.

**Statistical analysis**

No statistical methods were used to predetermine the sample size. Statistical tests, including two-tailed unpaired t-tests, two-tailed paired t-tests, and Pearson correlation tests were performed using GraphPad Prism (v9.0.0). For data presented without statistics, experiments were repeated at least three times independently.

**Supporting Reference**

1 Wang, Y. G. *et al.* KAT2A coupled with the alpha-KGDH complex acts as a histone H3 succinyltransferase. *Nature* **552**, 273-+ (2017). https://doi.org:10.1038/nature25003

2 Langmead, B. & Salzberg, S. L. Fast gapped-read alignment with Bowtie 2. *Nature Methods* **9**, 357-U354 (2012). https://doi.org:10.1038/Nmeth.1923

3 Li, H. *et al.* The Sequence Alignment/Map format and SAMtools. *Bioinformatics* **25**, 2078-2079 (2009). https://doi.org:10.1093/bioinformatics/btp352

4 Zhang, Y. *et al.* Model-based Analysis of ChIP-Seq (MACS). *Genome Biol* **9** (2008). https://doi.org:ARTN R13710.1186/gb-2008-9-9-r137

5 Ashburner, M. *et al.* Gene Ontology: tool for the unification of biology. *Nat Genet* **25**, 25-29 (2000). https://doi.org:Doi 10.1038/75556

6 Aleksander, S. A. *et al.* The Gene Ontology knowledgebase in 2023. *Genetics* **224** (2023). https://doi.org:10.1093/genetics/iyad031

7 Merico, D., Isserlin, R., Stueker, O., Emili, A. & Bader, G. D. Enrichment Map: A Network-Based Method for Gene-Set Enrichment Visualization and Interpretation. *Plos One* **5** (2010). https://doi.org:ARTN e1398410.1371/journal.pone.0013984

8 Shannon, P. *et al.* Cytoscape: A software environment for integrated models of biomolecular interaction networks. *Genome Res* **13**, 2498-2504 (2003). https://doi.org:10.1101/gr.1239303
